# Normalization of Tumor Vasculature by Oxygen Microbubbles with Ultrasound

**DOI:** 10.7150/thno.37750

**Published:** 2019-09-25

**Authors:** Yi-Ju Ho, Shu-Wei Chu, En-Chi Liao, Ching-Hsiang Fan, Hong-Lin Chan, Kuo-Chen Wei, Chih-Kuang Yeh

**Affiliations:** 1 Department of Biomedical Engineering and Environmental Sciences, National Tsing Hua University, Hsinchu, Taiwan.; 2 Institute of Bioinformatics and Structural Biology & Department of Medical Sciences, National Tsing Hua University, Hsinchu, Taiwan.; 3 Department of Neurosurgery, Chang Gung Memorial Hospital, Taoyuan, Taiwan.

**Keywords:** oxygen microbubbles, tumor perfusion, vascular normalization, oxygenation, ultrasound

## Abstract

Tumor microenvironment influences the efficacy of anti-cancer therapies. The dysfunctional tumor vasculature limits the efficiency of oxygenation and drug delivery to reduce treatment outcome. A concept of tumor vascular normalization (VN), which inhibits angiogenesis to improve vessel maturity, blood perfusion, and oxygenation, has been demonstrated under the anti-angiogenic therapy. The efficiency of drug delivery and penetration is increased by enhancing perfusion and reducing interstitial fluid pressure during the time window of VN. However, anti-angiogenic agents only induce transient VN and then prune vessels to aggravate tumor hypoxia. To repair tumor vessels without altering vessel density, we proposed to induce tumor VN by local oxygen release via oxygen microbubbles with ultrasound. With tumor perfusion enhancement under ultrasound contrast imaging tracing, the time window of VN was defined as 2-8 days after a single oxygen microbubble treatment. The enhanced tumor oxygenation after oxygen microbubble treatment inhibited hypoxia inducible factor-1 alpha (HIF-1α)/vascular endothelial growth factor (VEGF) pathway to improve the morphology and function of tumor vasculature. The pericyte coverage and Hoechst penetration of tumor vessels increased without any changes to the vessel density. Finally, the intratumoral accumulation of anti-cancer drug doxorubicin could be increased 3-4 folds during tumor VN. These findings demonstrate that regulating tumor oxygenation by oxygen microbubbles could normalize dysfunctional vessels to enhance vascular maturity, blood perfusion, and drug penetration. Furthermore, ultrasound perfusion imaging provides a simple and non-invasive way to detect the VN time window, which increases the feasibility of VN in clinical cancer applications.

## Introduction

Tumor oxygenation regulates cellular metabolism to influence the malignancy of the tumor microenvironment. Since the proliferation rate of tumor cells is faster than angiogenesis, the inadequate oxygen supply from blood circulation causes the tumor microenvironment to become hypoxic. In order to compensate for adequate oxygen delivery, increased expression of hypoxia inducible factor-1 alpha (HIF-1α) activates tumor cells to secrete vascular endothelial growth factor (VEGF) for angiogenesis 1. However, the overexpression of VEGF not only promotes neovascularization, but also aggravates vascular abnormity 2. The immature tumor vessels with leaky arrangement of endothelial cells, absent pericyte coverage, and incomplete basement membrane contribute to structural and functional abnormalities of the vascular network. Although tumor vascular density is promoted by angiogenesis, the irregular distribution of vasculature and heterogeneous blood perfusion restricts the efficiency of oxygen transport and impedes tumor reoxygenation 3. Under hypoxic conditions, overexpression of HIF-1α changes the proteome and genome of tumor cells, causing the tumor microenvironment to contribute to treatment resistance and metastasis 4. Hence, the HIF-1α/VEGF pathway regulates the deterioration of tumor microenvironments including oxygenation, vessel maturity, blood perfusion, and hypoxia to restrict the efficacy of tumor therapy.

In the last two decades, there is a growing interest in regulating the tumor microenvironment to decrease malignancy and improve treatment outcome 5. The concept is that “vascular normalization (VN)” remodels tumor vasculature into normal and mature phenotype under the anti-angiogenic status, which repairs vessel function to enhance blood perfusion and oxygenation 6. Bevacizumab (Avastin®), a clinical anti-angiogenic agent, has been used to promote endothelial cell arrangement tightening, pericyte coverage, and blood perfusion for normalizing tumor vasculature at 1-4 days after treatment 7, 8. Since anti-angiogenic agents only induce transient VN and then prune vessels to aggravate tumor hypoxia, the tumor oxygenation, vessel morphology, or blood perfusion should be traced over time to define the normalized window for combined therapies 9. Previous studies have combined chemo- or radio-therapy in the VN time window to improve the treatment outcome by the increased efficiency of drug delivery and tumor oxygenation because of the enhanced tumor perfusion 7, 8, 10. Furthermore, regulating the upstream or downstream genes of VEGF, like prolyl hydroxylase domain-containing protein 2 (PHD2), angiopoietin-1, and epidermal growth factor receptor, also provides various pathways for accomplishing VN 11.

**Figure 1:**
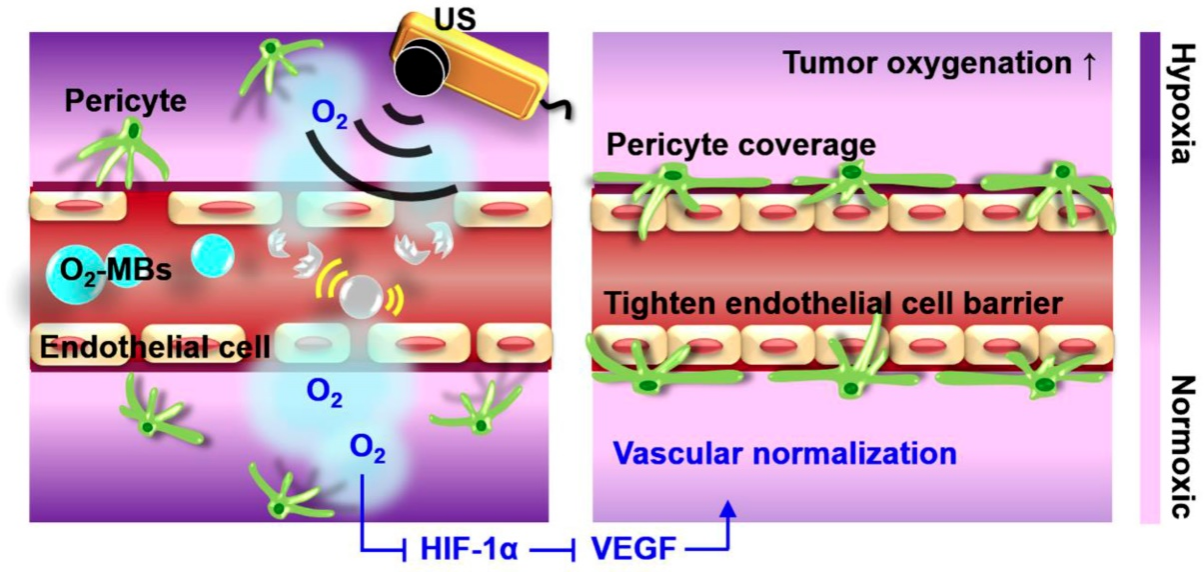
The concept of tumor VN induced by US with O_2_-MBs. The local oxygen release within tumors during O_2_-MBs treatment enhanced oxygenation and inhibited hypoxia/angiogenesis pathway to accomplish VN.

Oxygen plays an essential role in cellular function and metabolism. Microbubbles comprising a lipid shell and gaseous core can enhance the contrast of ultrasound (US) imaging and deliver various therapeutic gases or drugs for tumor theranosis applications 12, 13. Previous studies have used oxygen microbubbles (O_2_-MBs) to locally release oxygen into tumors by external US stimulation, and then improve therapeutic efficacy by reducing hypoxic treatment resistance 14-18. Since the elevated oxygenation decreases HIF-1α expression, the downstream factor VEGF would be inhibited to restrict tumor angiogenesis and deterioration 19. Therefore, we speculated that the promotion of tumor oxygenation by US-mediated O_2_-MBs destruction might regulate the expression of HIF-1α/VEGF pathway to accomplish VN (Figure 1). The feasibility of VN for murine prostate cancer induced by local oxygen release was evaluated to define the VN inducible dose of O_2_-MBs and corresponding normalized time window. In contrast to anti-angiogenic agents, VN induced by O_2_-MBs treatment might repair the morphology and function of tumor vessels without decreasing vascular density. In addition, tumor perfusion traced by ultrasound contrast imaging provides a potential and convenient way to evaluate the time window of VN for assisting clinical cancer therapy.

## Materials and Methods

### Characteristics of O_2_-MBs

To modify the optimal fabrication of O_2_-MBs, the gas mixture with various volume ratios of perfluoropropane (C_3_F_8_) and oxygen was infused into the phospholipid solution. The phospholipid solution in a sealed vial was composed of 2.5 mg of 1,2-Distearoyl-sn-glycero-3-phosphorylcholine (DSPC) and 1 mg of 1,2-distearoyl-sn-glycero-3-phosphoethanolamine-N-[10-(trimethoxysilyl)undecanamide(polyethylene glycol)-2000] (DSPE-PEG2000; Avanti Lipids Polar, Alabaster, AL, USA) in 0.8 mL of glycerol-phosphate-buffered saline (PBS, 5 wt%). The phospholipid solution was degassed and infused with the gas mixture with various volume ratios of perfluoropropane (C_3_F_8_; ABCR GmbH, Karlsruhe, Germany) and oxygen. Then, O_2_-MBs were fabricated via 45 seconds of violent shaking by an agitator (VIALMIX, Bristol-Myers Squibb Medical Imaging, New York, NY, USA). The additional C_3_F_8_ was used to improve the stability of O_2_-MBs due to the lower water solubility. In contrast, C_3_F_8_-MBs were made by the infusion with pure C_3_F_8_ gas to be the non-O_2_ MBs.

The concentration and size distribution of O_2_-MBs were detected by a coulter counter (Multisizer 3, Beckman Coulter, Miami, FL, USA). In order to estimate the amount of oxygen encapsulated within O_2_-MBs, the oxygen partial pressure (pO_2_) within the O_2_-MBs emulsion (1×10^7^ O_2_-MBs) was measured by a fiberoptic pO_2_ probe with an OxyLite 2000 system (Oxford Optronics, Oxford, UK). The oxygen release from O_2_-MBs was triggered by a sonication (2510, Branson, Danbury, CT, USA) for 5 min at 37 °C to totally destruct O_2_-MBs. To detect the stability of O_2_ within O_2_-MBs, the pO_2_ levels of O_2_-MBs in the PBS, degas PBS, and O_2_-PBS without US sonication were measured 17. The PBS solution was degassed for 3 min and infused O_2_ for 1 min to prepare O_2_-PBS. During O_2_ infusion, the needle was immersed into PBS to observe the bubble production.

### Acoustic characteristics of O_2_-MBs

The acoustic characteristics were measured to evaluate the difference between C_3_F_8_-MBs and O_2_-MBs. The contrast enhancement of US imaging and changes of pO_2_ levels were calculated to estimate the *in vitro* stability of MBs. Diluted MBs emulsion (concentration of 5×10^6^ MBs/mL) was infused into a cylindrical hollow chamber within the 3% agarose phantom at 37 °C. A fiberoptic pO_2_ probe with an OxyLite 2000 system was inserted into a cylindrical hollow chamber to detect the changes of pO_2_ levels. The US images were recorded over time with a US imaging system (7-MHz, Model t3000, Terason, Burlington, MA, USA) and the contrast-to-noise ratio produced from the MBs was analyzed. Since the violent oscillation and destruction of MBs might cause vessel damage, the* in vitro* destruction threshold and inertial cavitation dose (ICD) of MBs were evaluated to establish the safety US parameters for the subsequent local oxygen release. A 2-MHz high-intensity focused US (HIFU; model SU-101, Sonic Concepts, Bothell, WA, USA; diameter of 35 mm, focus length of 55 mm, focal size of 1.2 mm width × 13.3 mm depth) sonication system was used to stimulate O_2_-MBs (concentration of 5×10^6^ MBs/mL) in a PE-50 tube (Sunpoint scientific Instrument Co, Ltd, Taipei, Taiwan) with the flow rate of 6 mL/h. The 3-cycle US pulses with 2.25 Hz pulse repetition frequency (PRF) were used to stimulate MBs for measuring the destruction threshold and ICD under various peak negative pressures. The destruction threshold of MBs was evaluated by relative contrast enhancement of US images after US sonication. Moreover, a 15-MHz US transducer was used to receive the broadband noises, which were analyzed to calculate the ICD.

### Animal

All animal experiments were approved by the animal experiment committee at National Tsing Hua University (approval number: 10633). The animal procedures were performed following the guidelines of the Institutional Animal Care Committee. C57BL/6JNarl mice (male, 6-10 weeks old) were intraperitoneally anesthetized with a 1:1 mixture of Rompun 2% (Bayer HealthCare, Leverkusen, Germany) and Zoletil 50 (Virbac, Carros, France).

### *In vivo* vascular bioeffects

Since the ICD produced by O_2_-MBs violent oscillation and destruction might cause vessel damage, a mouse dorsal skinfold window chamber model (SM100, APJ Trading, Ventura, CA, USA) was used to observe the *in vivo* vascular bioeffects induced by US-stimulated O_2_-MBs destruction 20, 21. An acousto-optical system comprising a 2-MHz HIFU sonication system and an inverted microscope (IX71, Olympus, Tokyo, Japan) was used to recorded intravital images. Window chamber-mice were intravenously injected with 2×10^7^ O_2_-MBs and then stimulated by a 3-cycle single pulsed US with various peak negative pressures to observe the vessel response. The intravital images before and after US stimulation were collected to evaluate the safety parameters of the HIFU sonication system for O_2_-MBs destruction, which could locally release oxygen without vascular damage.

### Cell line and tumor model

The transgenic adenocarcinoma mouse prostate (TRAMP) cell line was cultured in Dulbecco's modified Eagle's medium supplemented with 1% penicillin-streptomycin and 10% fetal bovine serum (Gibco, Grand Island, NY, USA). Approximately 1×10^6^ TRAMP cells in 100 μL PBS were subcutaneously injected into the right leg of each mouse for tumor growth. Tumors were traced until they became established and reached a mean tumor volume of approximately 100-200 mm^3^.

### Local oxygen release within tumors

The integrated US image-guided treatment system was used to stimulate O_2_-MBs for local oxygen release within tumors 22. When the tumor volume reached 100-200 mm^3^ (day 0), the treatment dose of 2×10^7^ O_2_-MBs (50 μL) was retro-orbitally injected into mice. A 2-MHz HIFU transducer (2 MPa, 1000-cycle, PRF of 2 Hz) was moved manually by a triaxial platform at 0.5 mm intervals to finish the whole tumor scanning without heating 22. The acoustic pulse duration was prolonged to decrease the intensity attenuation in solid tumors. The total treatment time for whole tumor sonication was about 30 min. A fiberoptic pO_2_ probe was inserted into tumors to detect oxygen release kinetics within tumors during O_2_-MBs treatment by an OxyLite 2000 system. In order to confirm the possible biological damage after O_2_-MBs treatment, tumors (without pO_2_ probe insertion) were removed after 24 h and stained by hematoxylin and eosin (H&E) to evaluate tissue hemorrhage and necrosis.

### Tumor perfusion tracing

Since tumor perfusion is a potential biomarker for the occurrence of VN 23, 24, US contrast imaging with C_3_F_8_-MBs infusion was recorded to trace the relative perfusion intensity of whole tumors over time after O_2_-MBs treatment. To maintain the concentration of C_3_F_8_-MBs within the vasculature during whole tumor scanning, C_3_F_8_-MBs (2×10^9^ /mL) were infused by an injection pump with a velocity of 0.3 mL/h. The whole tumor perfusion images were collected with an interval of 0.5 mm. The enhanced signal between pre- and post-C_3_F_8_-MBs injection images was quantified to evaluate the tumor perfusion intensity and then normalized to day 0. The VN time window was defined when the tumor perfusion intensity was both higher than the control group and the baseline at day 0. Perfusion density was calculated as the area with enhanced signal in tumors to evaluate the density of the functional vessels. Moreover, the length, width, and depth of tumors were measured on two orthogonal US images to calculate and trace tumor volume.

### *In vivo* microdialysis analysis

Intratumoral oxygen release by US-mediated O_2_-MBs destruction could improve tumor oxygenation and reduce hypoxia. To verify the correlated gene pathway between oxygenation, angiogenesis, and cell proliferation, the variability of VEGF and transforming growth factor-beta (TGF-β) expression within tumors were traced. The *in vivo* microdialysis analysis was used to collect intratumoral tissue fluid at day 0, 2, 4, 6, and 8 after O_2_-MBs treatment. Microdialysis provided a non-sacrificed long-time protein collection in the same mice, which could trace the changes of microenvironment in tumors. The microdialysis probe (MAB 5, Microbiotech, Stockholm, Sweden) was implanted into the center of the tumor and collection was delayed for 30 min to reduce the interference from potential tumor damage. During dialysate collection, a probe was perfused with saline by a microinjection pump (CMA 402, CMA, Stockholm, Sweden) for 30 min (flow rate of 2.0 mL/h). Then, the protein concentrations of VEGF and TGF-β were analyzed in the microdialysis samples using the commercial ELISA kits (Rockland, Pennsylvania, USA) according to the manufacturer's protocols. After reaction, samples were measured using a plate reader system (Tecan Infinite M200, Tecan Trading AG, Switzerland) at an optical density of 450 nm to quantify the concentration of VEGF and TGF-β. The concentration of VEGF and TGF-β at each time point was normalized to that measured at day 0.

### Intratumoral protein expression during VN time window

Western blot analysis was used to detect the protein expression in whole tumors during VN time window, the time point was based on the significant perfusion enhancement and VEGF reduction. Tumors (*N* = 15) were removed at day 4 after O_2_-MBs treatment to confirm the protein expression during the period of perfusion enhancement. The collection obtained more proteins by homogeneous tissue than tissue fluid to suitably analyze various proteins expression. Tumor tissue was mechanically disrupted with RIPA lysis buffer (APOLLO, CA, USA). The samples were centrifuged at 13,000 rpm for 30 min at 4 °C. Proteins in the supernatants were quantified by Coomassie Protein Assay Reagent (BioRad Laboratories, Hercules, CA, USA). We diluted 30 μg of proteins in Laemmli sample buffer (pH 6.8, 50 mM Tris, 10% (v/v) glycerol, 2% SDS (w/v), 0.01% (w/v) bromophenol blue) and separated proteins by 1D-SDS-PAGE. The proteins were separated onto 0.45 μm Immobilon P membranes (Millipore, Bedford, MA, USA) and the membranes were immersed in a blocking solution with 5% w/v skim milk in TBS-T (50 mM Tris pH 8.0, 0.1% Tween-20 (v/v), and 150 mM NaCl) for 1 h. After that, membranes were incubated with primary antibodies (1: 2000; GeneTex, Irvine, CA, USA) of HIF-1α, VEGF, TGF-β, and PHD2 overnight at 4 °C and washed 4 times with TBST (150 mM NaCl and 0.1% Tween 20 in 10 mM Tris-HCL, pH 7.4) for 10 min. The horseradish peroxidase-conjugated AffiniPure Goat Anti-Rabbit IgG (H+L) (1:10000; Jackson ImmunoResearch, West Grove, PA, USA) was added at room temperature for 1 h, and then membranes were washed 6 times with TBST for 10 min. Finally, membranes were visualized by an enhanced chemiluminescence method (Visual Protein Biotechnology, Taipei, Taiwan) and the expression levels of the proteins were measured by an Image-Quant TL software (GE Healthcare Bio-Sciences, Uppsala, Sweden).

### Tumor immunohistochemistry

The modulation of tumor microenvironment was confirmed by assessing the function and morphology of vasculature by immunohistochemistry. At day 4 after O_2_-MBs treatment, mice were intravenously injected with Hoechst 33342 (15 mg/kg; Invitrogen, Carlsbad, CA, USA) and the dye was allowed to circulate for 1 min before the mouse was sacrificed and perfused with 0.9% normal saline to remove the Hoechst from the circulation. The Hoechst stain was used to evaluate the diffusion function of vessels. Furthermore, 20-μm-thick tumor sections were stained for endothelial cells and pericytes using rat anti-mouse CD31 (1: 100; GeneTex, Irvine, CA, USA) and mouse monoclonal alpha-smooth muscle actin (α-SMA) directly conjugated with fluorescein isothiocyanate secondary antibody (1: 100; GeneTex, Irvine, CA, USA). Tumors were separated into three equal parts, with each part defined as central (inner half-radii) or peripheral regions (outer half-radii); the three random fields per tumor region were analyzed. Each tumor had a total of 18 fields for histological quantification: (1) vessel density: density of endothelial cells; (2) vessel diameter: size of tumor vessels; (3) vascular maturity: ratio of pericyte coverage on endothelial cells (α-SMA/CD31); (4) Hoechst intensity: the fluorescence intensity of Hoechst per field; (5) Hoechst leakage: quantification of Hoechst intensity normalized to vessel density 25, 26; (6) Hoechst penetration: the fluorescence intensity curve of Hoechst from the vessel wall to the tumor tissue.

### Clinical anti-cancer drug accumulation

In this study, we used chemotherapeutic drug doxorubicin (DOX) to confirm the improvement of drug accumulation within tumors after enhancing tumor perfusion by VN. Tumor-bearing mice were separated into the control, C_3_F_8_-MBs+US, and O_2_-MBs+US groups. After MBs treatment 4 days, mice were intravenously injected DOX (7 mg/kg) without MBs infusion. After 24 h, tumors were removed to evaluate the intratumoral drug distribution by histological assessment. The red-fluorescence intensity of DOX in histological images was analyzed using MATLAB (MathWorks, Natick, MA, USA) software to quantify the accumulation of DOX in solid tumors. The quantification of microscopic images was followed the above procedure of immunohistochemistry analysis.

### Statistical analysis

Results were presented as mean ± standard deviation. In each graph, the error bars indicated the standard deviation. For *in vivo* experiments, four to eight mice were recruited to each group. The multiple comparisons were made by one-way ANOVA (SPSS 13.0, SPSS Inc., IBM, Armonk, NY, USA), and the statistical significance was defined as *p*-value less than 0.05.

## Results

### Characteristics of O_2_-MBs

**Figure 2:**
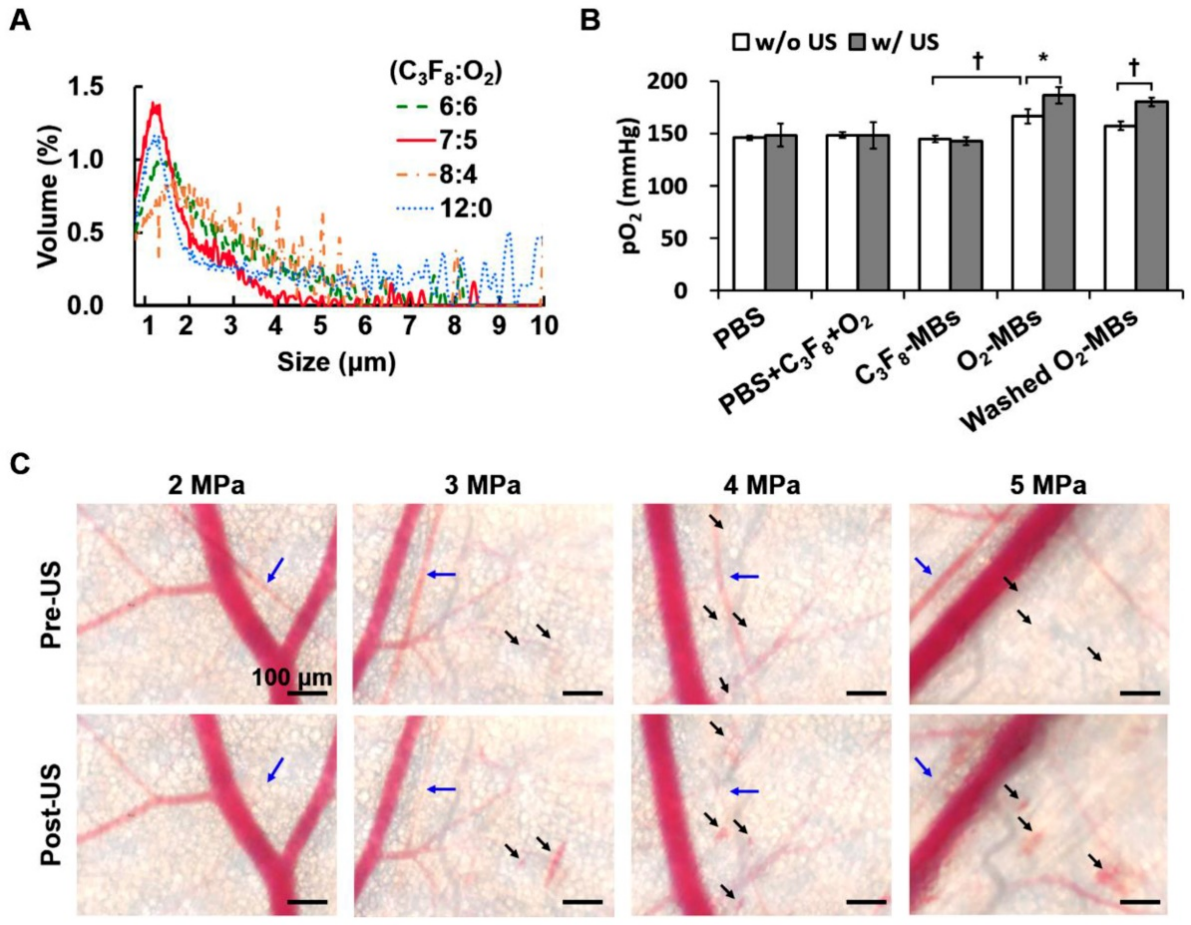
(A) Volume distribution of O_2_-MBs with various volume ratios of C_3_F_8_ and O_2_. (B) The pO_2_ levels within O_2_-MBs emulsion (C_3_F_8_:O_2_=7:5). Oxygen release was triggered by sonication for 5 min at 37 °C. The legends of w/o and w/ mean without US and with US sonication, respectively. (C) The intravital images were collected to evaluate the *in vivo* vascular bioeffects induced by O_2_-MBs destruction. A mouse dorsal skinfold window chamber model was placed on an acousto-optical system. Intravital images revealed the vascular constriction (→) and disruption (➝) during O_2_-MBs destruction under various acoustic pressures. Scale bar, 100 μm. Quantitative results are presented as mean ± standard deviation and were analyzed by one-way ANOVA (* *p*<0.05; † *p*<0.01).

Different volume ratios of C_3_F_8_ and O_2_ were encapsulated in the lipid-shelled O_2_-MBs. Based on the smaller size and higher concentration than other groups, the optimal fabrication of O_2_-MBs was made by a C_3_F_8_:O_2_ volume ratio of 7:5 (Figure 2A and Figure S1A). The mean size and concentration of O_2_-MBs (C_3_F_8_:O_2_=7:5) were 1.02±0.03 μm (mean±standard deviation) and (20.54±3.46) ×10^9^ MBs/mL, respectively. The pO_2_ levels within the O_2_-MBs emulsion increased 21±7 mmHg relative to the PBS, and then O_2_-MBs released oxygen in a sonication bath to promote pO_2_ levels from 166±7 to 186±8 mmHg (*p*<0.05; Figure 2B). In order to demonstrate that the enhanced pO_2_ level was from O_2_-MBs not buffer, O_2_-MBs was centrifuged 2000 rcf for 1 min and washed by PBS, called washed O_2_-MBs. The pO_2_ levels showed no significant difference between O_2_-MBs and washed O_2_-MBs which indicated that O_2_ was mostly encapsulated within O_2_-MBs. Besides, the pO_2_ levels of 1×10^7^ O_2_-MBs in the PBS and O_2_-PBS showed more stable than that in the degas PBS, which demonstrated the loading stability of O_2_ within O_2_-MBs (Figure S2).

To evaluate the acoustic features of the O_2_-MBs, the* in vitro* stability, destruction threshold, and ICD were measured. The contrast enhancement of US images and pO_2_ levels by C_3_F_8_-MBs and O_2_-MBs revealed no significant reduction after 60 min at 37 °C (Figure S1B). A 2-MHz HIFU with an acoustic pressure of 2 MPa showed 100% MBs destruction, which was used to stimulate O_2_-MBs destruction and trigger local oxygen release (Figure S1C). In order to evaluate the possible bioeffects induced by the mechanical force from the MBs destruction, the ICD was measured, providing a direct proportion to the acoustic pressures in the C_3_F_8_-MBs and O_2_-MBs (*p*>0.05; Figure S1D). Since ICD might have an anti-vascular effect that reduces tumor perfusion for anti-vascular therapy 20, we used a mouse dorsal skinfold window chamber model to confirm the vascular bioeffects during O_2_-MBs destruction. The intravital images revealed vasoconstriction after US stimulation with 2 MPa and showed vascular disruption under 3 to 5 MPa (Figure 2C). According to the above-mentioned results, US-mediated O_2_-MBs destruction could increase the pO_2_ without inducing vascular bioeffects under the acoustic pressure of 2 MPa.

### Tumor perfusion enhancement during VN

**Figure 3:**
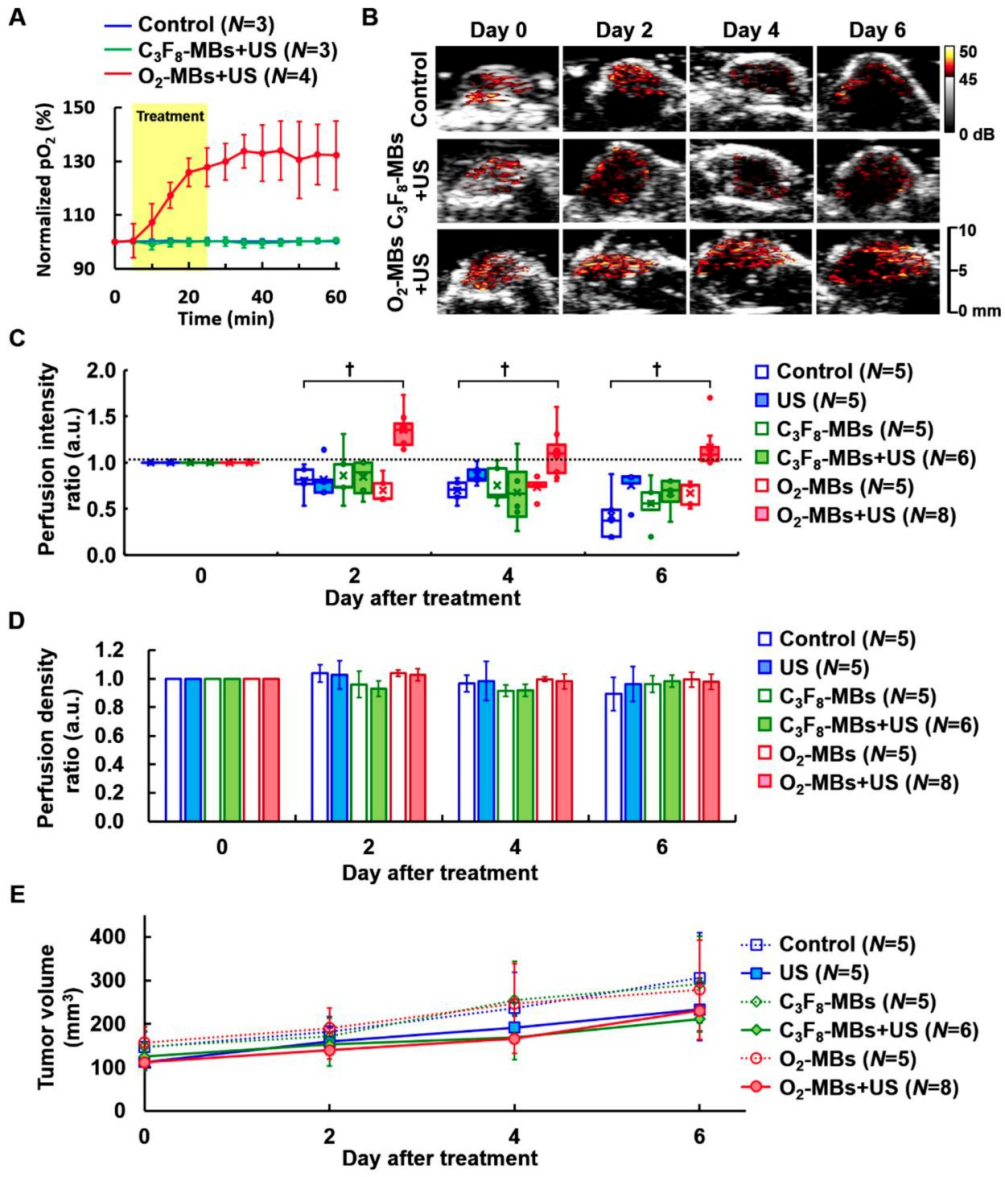
(A) The normalized intratumoral pO_2_ was significantly increased by O_2_ release from O_2_-MBs during US sonication. (B) The blood perfusion of TRAMP tumors was traced over time by US contrast imaging. The infusion of C_3_F_8_-MBs (2×10^9^ /mL) displayed the enhanced signal on US imaging to estimate the perfusion ability. (C) The perfusion intensity ratios were relative to day 0 of each group, which were traced over time after various treatment protocols. The treatment dose of C_3_F_8_- or O_2_-MBs was 2×10^7^ per mouse. Data are presented as box-and-whiskers plots, each dot represents a sample (*N*=5 to 8 mice per group). (D) Perfusion density ratios showed no difference in the O_2_-MBs+US group. (E) Tumor volume was traced to compare the growth rate between each group (*p*>0.05). Data are presented as mean ± standard deviation and were analyzed by one-way ANOVA (* *p*<0.05; † *p*<0.01).

To study the impact of local oxygen release from O_2_-MBs on tumors, we used TRAMP tumors to evaluate the feasibility of VN. The normalized intratumoral pO_2_ was significantly increased and maintained 132±13% over 30 min after O_2_-MBs treatment (Figure 3A). The intratumoral pO_2_ levels were increased from 16±4 to 22±6 mmHg at 0 to 60 min (Figure S3). In Figure 3B, the perfusion images from the maximum section of the tumor show higher perfusion intensity in the O_2_-MBs+US group than in the control and C_3_F_8_-MBs+US groups, especially at day 4 and 6 after treatment. Quantification of tumor perfusion showed an enhanced signal of C_3_F_8_-MBs on US images. The perfusion intensity ratio in the O_2_-MBs+US group was 1.00±00, 1.35±0.20, 1.10±0.27, and 1.16±0.24 at day 0, 2, 4, and 6, respectively (Figure 3C). In comparison to the control group, the perfusion density showed no significant reduction by the O_2_-MBs treatment (Figure 3D). Although angiogenesis is promoted during tumor growth, the vessel density in the control group showed no significant difference due to the tumor volume increasing 7, 27-29. Since the O_2_-MBs treatment did not influence the vessel density of the tumors, the subsequent hypoxia aggravation would be mitigated to improve the treatment outcome. These results further confirm that tumor perfusion is enhanced through the repair of vessel function during VN instead of angiogenesis. The trace of tumor volume showed no significant difference in growth rate between the groups, demonstrating that increased tumor oxygenation does not accelerate tumor cell proliferation (Figure 3E).

**Figure 4:**
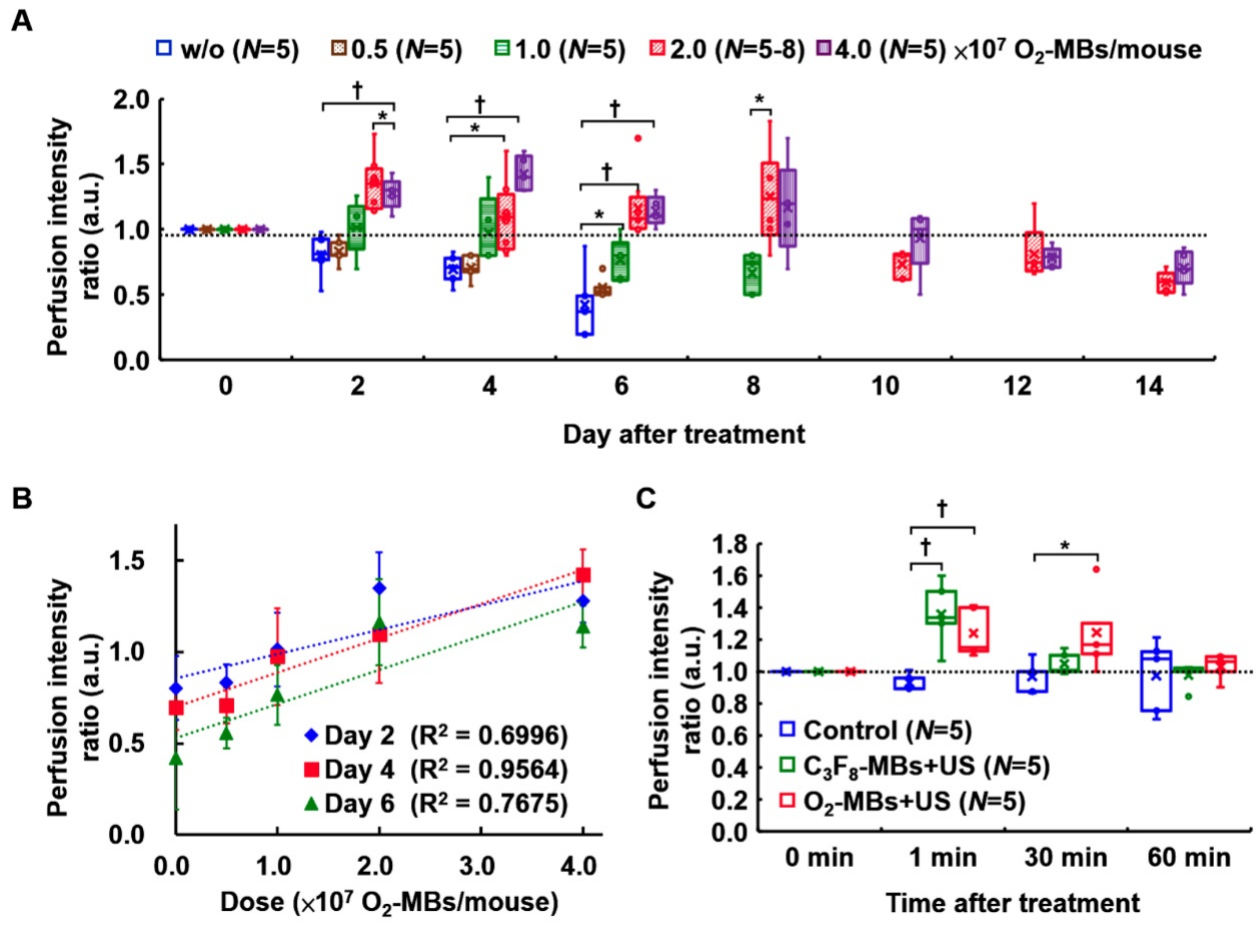
(A) The threshold of tumor perfusion enhancement at various O_2_-MBs doses was evaluated to determine the VN inducible dose of O_2_-MBs. The legend of w/o means without O_2_-MBs injection. (B) Linear correlation between perfusion intensity ratio and O_2_-MBs dose. The correlation coefficients are 0.659, 0.795, and 0.719 at day 2, 4, and 6 after O_2_-MBs treatment, respectively. Data are presented as mean ± standard deviation. (C) The transient effect of perfusion enhancement by MBs cavitation. Data are presented as box-and-whiskers plots and analyzed by one-way ANOVA (* *p*<0.05; † *p*<0.01). Each dot represents a sample (*N*=5 to 8 mice per group).

The dose dependence of tumor perfusion enhancement by O_2_-MBs treatment was also investigated in the present study. In the control group, the end point of perfusion tracing was at day 6 because the subsequent tumor perfusion was always lower than day 0 and decreased over time. In a comparison of the perfusion intensity ratio of the control tumors (w/o O_2_-MBs injection), the dose of 0.5×10^7^ O_2_-MBs showed no significant difference from day 2 to day 6 (each *p*>0.05; Figure 4A). The dose of 1.0×10^7^ O_2_-MBs displayed perfusion enhancement at day 6 relative to the control group (*p*<0.05), which was lower than that at day 0. Notably, tumor perfusion relative to day 0 in the 2.0 and 4.0×10^7^ O_2_-MBs groups maintained the enhancement until day 8 after treatment, but the difference between doses is not significant. At day 4 after treatment, the doses of 0, 0.5, 1.0, 2.0, and 4.0×10^7^ O_2_-MBs per mouse showed perfusion intensity ratios of 0.70±0.12, 0.71±0.10, 0.97±0.27, 1.10±0.27, and 1.43±0.14, respectively. The correlation coefficient for the relationship between the O_2_-MBs dose and perfusion intensity ratio was 0.659, 0.795, and 0.719 at day 2, 4, and 6 after treatment, respectively (Figure 4B). Therefore, the VN inducible dose of O_2_-MBs was 2.0×10^7^ O_2_-MBs per mouse, with a normalized time window of 2-8 days.

Further, blood perfusion can be enhanced by MBs cavitation, where the increased shear stress on endothelial cells promotes nitric oxide generation and induces vasodilation 30. The transient effect of perfusion enhancement by MBs cavitation is shown in Figure 4C. However, this vascular enhancing effect by MBs cavitation only presents after 1-30 min and then returns to the baseline at 60 min. Thus, the long-term enhancement of tumor perfusion after O_2_-MBs treatment was induced by VN instead of MBs cavitation. The biological effects induced by MBs cavitation were also evaluated by tumor perfusion and histological images. Pervious study used non-recovery perfusion reduction to demonstrate the efficiency of anti-vascular effect induced by MBs cavitation 20, 31. In the present study, intravital images of vessel network on the mouse dorsal skin showed vasoconstriction at small vessels after US stimulation but there was no vessel disruption and hemorrhage (Figure 2C). The enhanced tumor perfusion at 1-30 min after O_2_-MBs treatment indicated the absence of anti-vascular effect (Figure 4C). Moreover, the histological images stained by H&E revealed intact tumor structure without hemorrhage and necrosis (Figure S4). So that, tumor vessel density showed no significant reduction by the O_2_-MBs treatment in comparison to the control group (Figure 3D). These results demonstrated that the slight inertial cavitation was produced by O_2_-MBs under 2 MPa US sonication and biological damage did not be observed.

### Regulation of protein expression after O_2_-MBs treatment

**Figure 5:**
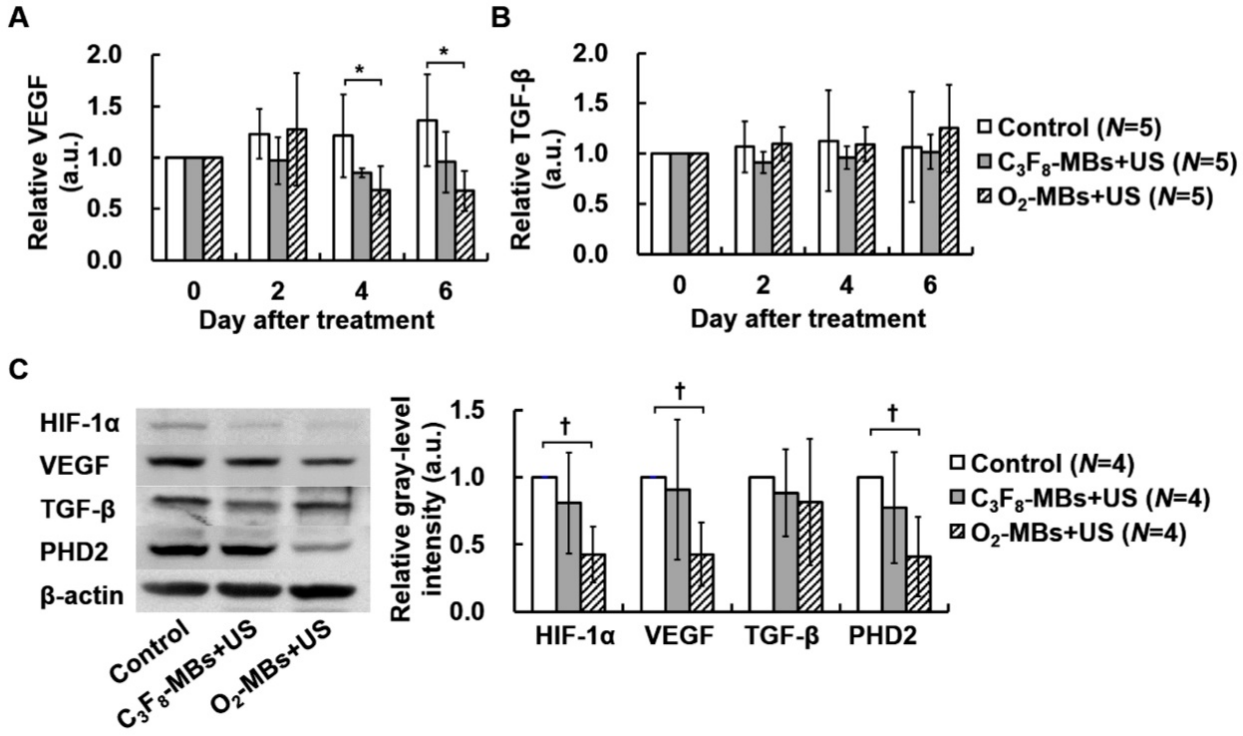
Variability in protein expression after O_2_-MBs treatment. The relative expressions of (A) VEGF and (B) TGF-β were traced over time by *in vivo* microdialysis and measured by ELISA assay. (C) Whole tumoral HIF1-α, VEGF, TGF-β, and PHD2 expression was determined by Western blot. Data are presented as mean ± standard deviation and were analyzed by one-way ANOVA (* *p*<0.05; † *p*<0.01).

To estimate the potential gene pathway after tumor VN induced by O_2_-MBs treatment, the intratumoral protein expression was measured. In the control tumors, the concentration of VEGF showed a non-significant increase of 0.16±0.03 pg/mL at day 0 to 0.22±0.08 pg/mL at day 6 (*p*>0.05; Figure S5A). The level of VEGF expression normalized to day 0 maintained consistent ratios in the control and C_3_F_8_-MBs+US groups; but started to significantly decrease to 0.68±0.24 at day 4 in the O_2_-MBs+US group (Figure 5A). The TGF-β expression was measured to evaluate the variability of cell proliferation under well oxygenation condition. The concentration and relative TGF-β expression presented no significant difference between each group, revealing consistent results of tumor volume tracing (*p*>0.05; Figure S5B and Figure 5B). Moreover, the protein expression in whole tumors by Western blot analysis was evaluated at day 4 after treatment because of the significant perfusion enhancement and VEGF reduction. There was a significant reduction of 0.43±0.21, 0.43±0.24, and 0.41±0.26 for HIF-1α, VEGF, and PHD2 expression, respectively, relative to control tumors (Figure 5C). The variability of intratumoral protein expression after O_2_-MBs treatment demonstrated that promoting tumor oxygenation can reduce tumor hypoxia to inhibit the expression of VEGF for VN.

### Repairing morphology and function of tumor vessels

**Figure 6:**
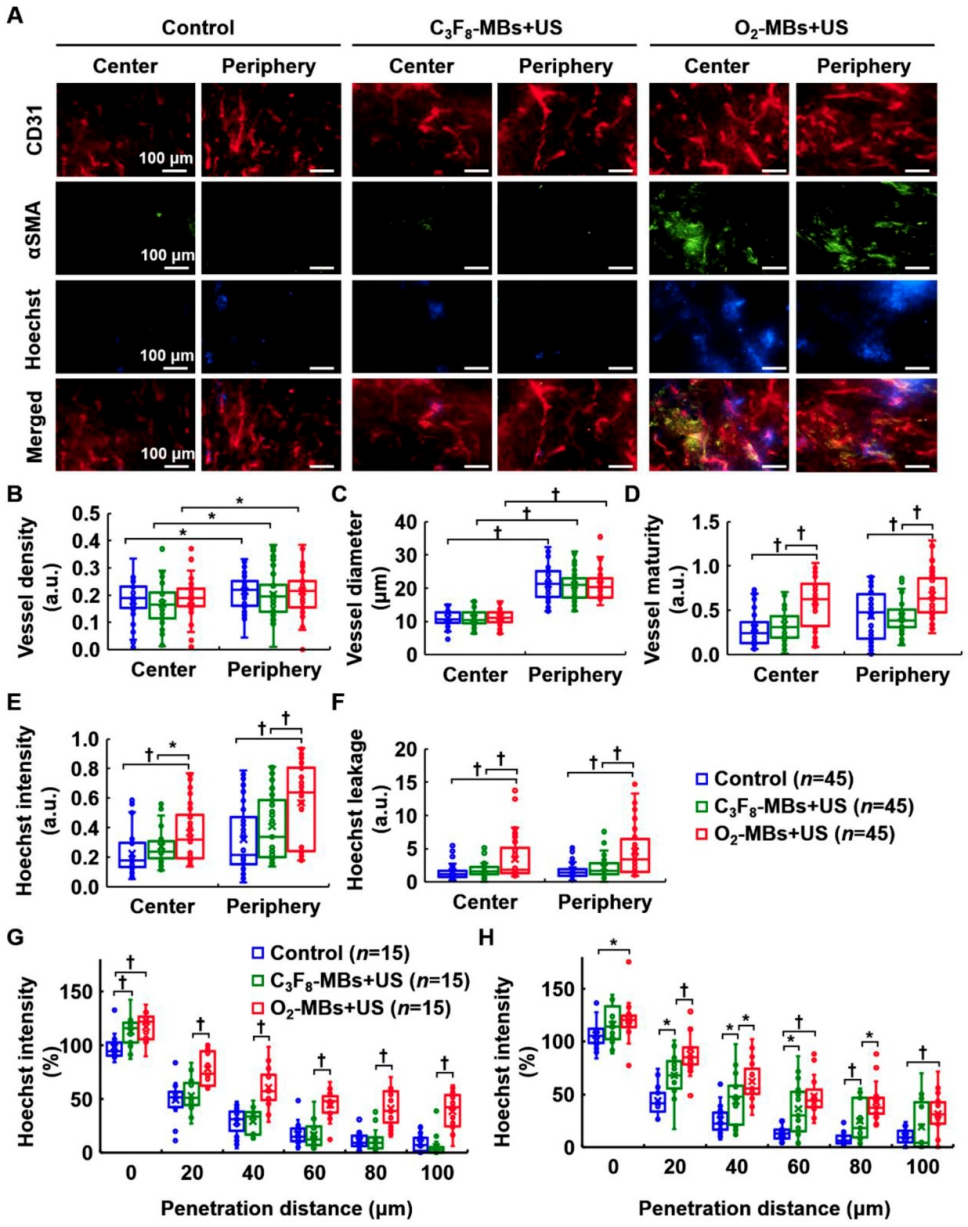
Histological assessment of tumor vascular morphology and function. (A) CD31 and αSMA staining indicated endothelial cells (red) and pericytes (green). The Hoechst dye (blue) was used to display the ability of vascular perfusion and permeability. Scale bar, 100 μm. Quantification results from histological images included (B) vessel density, (C) vessel diameter, (D) vessel maturity, (E) Hoechst intensity, and (F) Hoechst leakage. The distances of Hoechst penetration into the (G) tumor center and (H) tumor periphery were measured by fluorescence intensity curve from the vessel wall to the tumor tissue. Each group contained 5 mice (*N*=5). Images were analyzed from nine random fields per region in the tumor center or periphery. Data are presented as box-and-whiskers plots and analyzed by one-way ANOVA (* *p*<0.05; † *p*<0.01). Each dot represents an image (*n*=45).

To demonstrate the occurrence of tumor VN, the morphology and function of tumor vessels were evaluated at day 4 after local oxygen release. Histological images revealed more pericyte (αSMA) coverage on the endothelial cells (CD31) in the central and peripheral regions after O_2_-MBs treatment (Figure 6A). The extravascular leakage of Hoechst dye was presented to identify the repaired vessel function of perfusion and permeability in the O_2_-MBs+US group. Quantification showed no significant difference in the vessel density and diameter between each group (each *p*>0.05; Figure 6B and 6C). The vessel maturity of O_2_-MBs+US group increased 1.99±0.35- and 1.53±0.39-fold in the tumor central and peripheral regions, respectively, relative to the control tumors (each *p*<0.01; Figure 6D). Hoechst intensity was analyzed to evaluate the vascular perfusion of tumors, and the O_2_-MBs+US group revealed significant enhancement with the control and C_3_F_8_-MBs+US groups in the tumor center and periphery (each *p*<0.01; Figure 6E). The Hoechst leakage of the O_2_-MBs+US group increased 2.20±3.33- and 2.54±4.02-fold in the tumor central and peripheral regions relative to the control tumors (each *p*<0.01; Figure 6F). The increase in functional tumor vessels was caused by promoting the vascular maturity. Moreover, the distance of Hoechst penetration was significantly increased after O_2_-MBs treatment. The Hoechst intensity at a penetration distance of 80 μm was 11±8, 12±11, and 41±81% in the control, C_3_F_8_-MBs+US, and O_2_-MBs+US groups, respectively at the tumor center (Figure 6G), and 8±6, 25±20, and 43±19% at the tumor periphery (Figure 6H).

**Figure 7:**
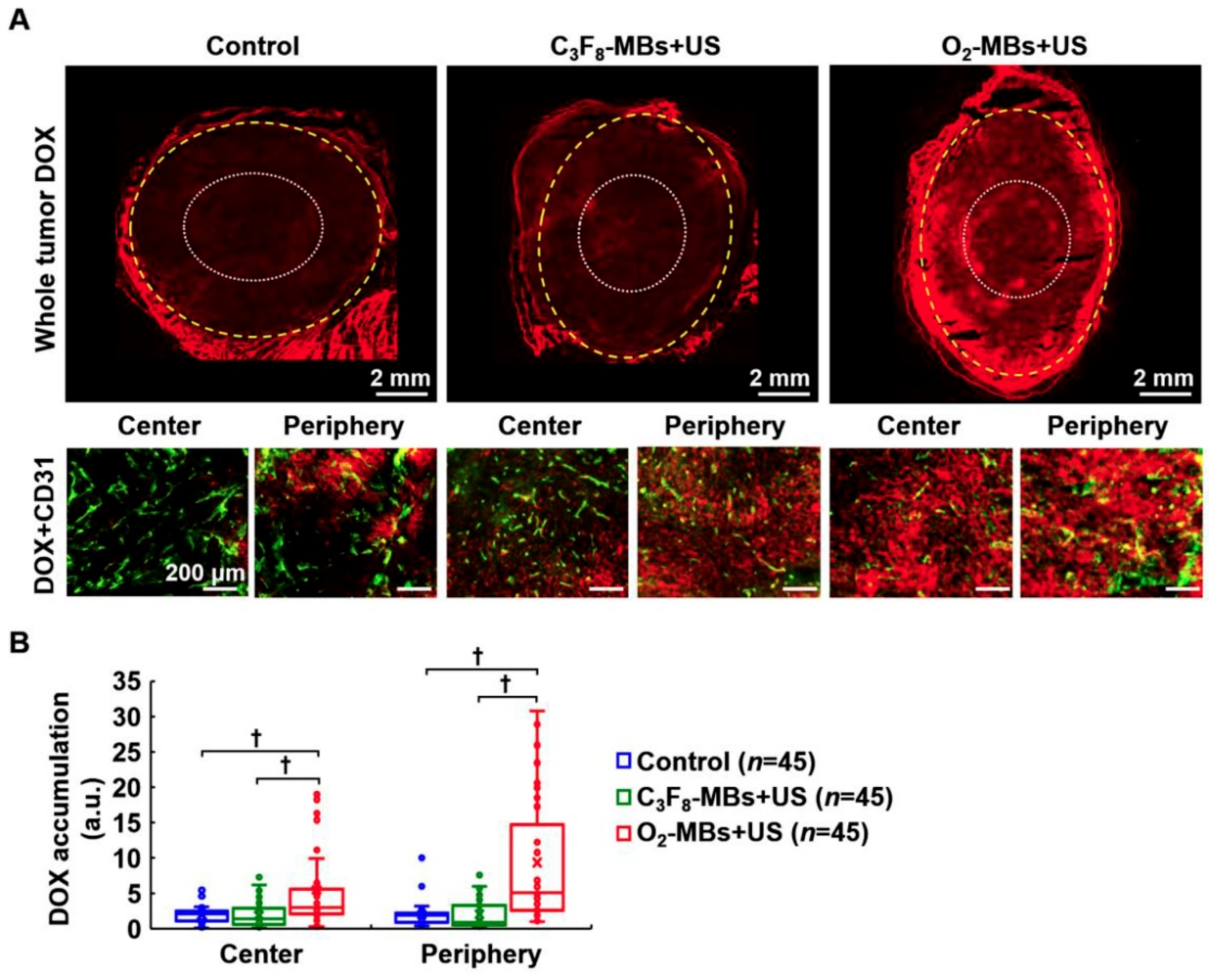
Evaluation of intratumoral drug accumulation. DOX (7 mg/kg) was injected into mice at day 4 after O_2_-MBs treatment. (A) Histological images for DOX (red) and CD31 staining (endothelial cells, green) show the distribution of drugs within the whole tumor, tumor center (white dotted line), and tumor periphery (yellow dashed line). (B) Quantification of intratumoral DOX accumulation. Each group contained 5 mice (*N*=5). Images were analyzed from nine random fields per region in the tumor center or periphery. Data are presented as box-and-whiskers plots and analyzed by one-way ANOVA (* *p*<0.05; † *p*<0.01). Each dot represents an image (*n*=45).

In order to demonstrate the efficiency of intratumoral drug accumulation improved by VN, the chemotherapeutic drug DOX was administrated at day 4 after O_2_-MBs treatment. Histological images showed more DOX distribution within the tumor core and periphery in the O_2_-MBs+US group (Figure 7A). The drug accumulation in the control, C_3_F_8_-MBs+US, and O_2_-MBs+US group was 1.96±1.12, 1.87±1.72, and 5.50±5.86, respectively, in the tumor center, and 2.01±1.75, 2.03±2.01, and 9.34±8.82, respectively, in the tumor periphery (Figure 7B). In the histological assessment, the enhancement of vessel maturity, Hoechst intensity, and Hoechst leakage demonstrated that dysfunctional tumor vasculature was restored by normalizing the morphology of the vessels. Moreover, the drug accumulation within tumors can also be improved by enhancing the efficiency of drug delivery during the VN time window.

## Discussion

The tumor vasculature provides gas exchange and molecular transport to influence the tumor microenvironment. Excessive angiogenesis causes distorted and leaky tumor vessels that reduce the efficiency of blood perfusion. Due to the porous vessel wall, intravascular plasma, molecular, and nano-sized particles passively penetrate into tumor tissue and are retained due to the dysfunctional lymphatic system, called the enhanced permeability and retention (EPR) effect 32. Although the EPR effect can increase the nanodrug accumulation within tumors, the elevated interstitial fluid pressure (IFP) restricts the penetration distance of nanodrugs, resulting in non-uniform drug distribution 33. Tumor VN can remodel abnormal tumor vessels into normal and mature phenotypes under anti-angiogenic status 6. The normalized tumor vasculature has a mature morphology to enhance blood perfusion through repair of vessel function, reduction of IFP by vessel pore shrinking, and prevention of hypoxia by promoting oxygenation, which improves the malignant tumor microenvironment and treatment outcome 34. Chauhan et al. proved that the intratumoral penetration was enhanced for the 12 nm nanoparticles after VN, but showed no improvement for the 60 and 125 nm nanoparticles 35. Although tumor VN might decrease the EPR effect by shrinking the vascular pore size, the decreased IFP could enhance the penetration of small-nanosized or molecular drugs to improve therapeutic efficacy 7, 28. The balance between tumor VN and the EPR effect was mainly associated with the size of drugs in drug penetration. Thus, we used small molecular drug DOX to exclude the interference of drug sizes and demonstrated the enhancement of drug penetration after tumor VN in the present study. Besides, the efficiency of tumor VN induced by O_2_-MBs treatment was associated with the initial tumor size. The initial tumor diameter of 8 mm reduced perfusion intensity over time after O_2_-MBs treatment, but tumor necrosis occurred according to the histological images staining by H&E (data was not shown). Thus, the initial tumor diameter to 6 mm (100-200 mm^3^) was used to investigate tumor VN in this study.

Tumor oxygenation influences HIF-1α expression to regulate the balance between pro- and anti-angiogenesis in neovasculature progression 34. In tumor hypoxic conditions, tumor cells secrete HIF-1α to activate VEGF expression for angiogenesis. Thus, improving tumor oxygenation could inhibit HIF-1α secretion, and then reduce the downstream VEGF protein level. In the present study, we proposed a potential pathway to induce VN by improving tumor oxygenation. The tumor perfusion enhancement, HIF-1α and VEGF expression reduction, and mature morphology of vessels after O_2_-MBs treatment demonstrated the feasibility of VN induced by local oxygen release. The increased penetration distance of Hoechst dye indicates the possibility of IFP reduction after VN. Since the improvement of intratumoral drug accumulation after VN shows high correlation with the size of nanodrugs, the small clinical anti-cancer drug DOX was used in our study and was shown to have more drug accumulation within tumors after O_2_-MBs treatment. Furthermore, US-mediated MBs cavitation has been widely applied to promote cellular and vascular permeability for drug and gene delivery 36, 37. However, these vascular enhancing effects only present short-term changes to assist with immediate drug delivery. The intratumoral distribution of drugs might be non-uniform and only accumulate around the vessel wall because the dysfunctional vessel network and high IFP have yet to be solved.

Previous studies used commercial US imaging system (mechanical index= 0.3-1.3) with MBs infusion to evaluate the possible vascular bioeffects for contrast enhanced sonography 38-40. MBs cavitation not only increases the intravascular shear stress to induce vasodilation, the intracellular hydrogen peroxide, calcium ion flux, and ATP generation are also increased to regulate the metabolism of endothelial cells 30, 41, 42. Besides, the augmentation of blood flow by MBs cavitation has been applied to provide a prevention way for ischemia-reperfusion injury after stroke, thrombolysis, surgery, and organ transplantation 30, 43, 44. In the present study, this transient enhancement of blood perfusion induced by C_3_F_8_-MBs treatment with therapeutic US (mechanical index=1.4) was also observed in the tumor vasculature. The enhanced tumor perfusion only maintained 30 min after C_3_F_8_-MBs treatment but revealed no significant difference in the tumor vascular morphology. The intratumoral drug penetration in the present study was investigated at day 4 after C_3_F_8_-MBs treatment. The penetration distance of hoechst within tumor periphery showed significant increment in the C_3_F_8_-MBs +US group relative to the control tumors. Notably, the time point of hoechst administration not accompanied with MBs injection. Thus, the enhanced penetration of hoechst was not caused by the increased vascular permeability during MBs cavitation. Although the mechanism of penetration improvement after C_3_F_8_-MBs treatment was not clear, the change in endothelial cell metabolism induced by the increased shear stress might be the potential reason.

An oxygen-sensor PHD2 regulates endothelial cell arrangement and pericyte coverage in angiogenesis to influence the maturity of vessels 45. Inhibition of PHD2 expression can reset oxygen sensing in tumors to adapt to hypoxia and induce VN 46-48. Besides, TGF-β expression is associated with tumor cell proliferation, which was used to evaluate whether the improvement of tumor oxygenation would affect tumor growth or not in our study. The expression of TGF-β also inhibited the degradation of HIF-1α by decreasing the protein levels of PHD2 49. Liu et al. blocked TGF-β to decrease HIF-1α and VEGF for inducing tumor VN in breast carcinoma 50. However, since TGF-β is not the downstream factors of HIF-1α, improving tumor oxygenation showed no significant difference in the protein levels of TGF-β. According to the upstream gene regulation, O_2_-MBs treatment reduces the level of HIF-1α to affect downstream VEGF and PHD2 expression. The mechanism of VN induced by O_2_-MBs treatment involves inhibition of HIF-1α, VEGF, and PHD2 expression to regulate multiple hypoxic and angiogenic genes. A previous study reported that dual inhibition of VEGF receptor and angiopoietin-2 could prolong the VN time window compared with VEGF receptor inhibition alone 51. Since the anti-angiogenic agent is used to inhibit vessel growth, the balance between pro- and anti-angiogenesis for inducing VN is transient 7, 8. The excessive pruning tumor vessels after VN time window reduced perfusion and increased hypoxia to cause probable treatment resistance and poor prognosis. On the other hand, O_2_-MBs-induced VN was regulated by hypoxic and angiogenic gene expression under tumor reoxygenation. The non-significant difference in vascular density after O_2_-MBs treatment might palliate the aggravation of tumor microenvironment, which might be another reason to prolong the VN time window.

Tumor VN can also reverse the immunosuppressive microenvironment because the enhanced tumor perfusion promotes the infiltration and activity of immune cells 52. The tumor microenvironment usually accumulates abundant myeloid-derived suppressor cells, tumor-associated macrophages, and regulatory T cells to evade host immunosurveillance 53. The hypoxic condition in tumors reduces the activity of immune cells and increases the recruitment of immune suppressors to contribute to immune suppression by activating HIF-1 and VEGF pathways 54. The low dose of anti-angiogenic treatment promotes the polarization of tumor-inhibitory M1-like tumor-associated macrophages and the infiltration of CD4^+^ and CD8^+^ T cells 55. Since the high IFP would compress the intratumoral lymphatic vessels, the reduction of IFP by VN might help antigen-presenting cells return to home to lymph nodes for immune activation. Moreover, VN induced by PHD2 inhibition prevented tumor cell migration through the porous vessel wall into the circulation 56, 57. The shrinking pore size of tumor vessels reduced the probability of tumor metastasis. Therefore, VN not only remodels defective tumor vasculature, but also repairs malignant tumor microenvironment to reverse immunosuppression and impede metastasis. In the future work, the possibility of prolonging the VN time window should be investigated by multiple injections of O_2_-MBs or by combination of anti-angiogenic agents and O_2_-MBs. The regulations of the tumor microenvironment for immunosuppression and metastasis should be discussed under the O_2_-MBs treatment.

In summary, we proposed that local oxygen release by US-mediated O_2_-MBs destruction regulates the tumor microenvironment to inhibit hypoxia and angiogenesis for accomplishing VN. Tumor vascular maturity and function were improved to enhance tumor perfusion during the VN time window of 2 to 8 days after O_2_-MBs treatment. And then, enhanced tumor perfusion promotes the efficiency of drug delivery for increasing intratumoral drug accumulation. In light of this view, US perfusion imaging could be applied to trace tumor perfusion *in vivo*, allowing us to evaluate whether VN is induced or not after O_2_-MBs or anti-angiogenic treatment. When tumor perfusion was higher than the first-time measurement, the time window of VN for subsequent chemo- or radio-therapy could be defined to establish personal medicine information. This convenient and easy approach of US perfusion imaging can be used as part of clinical diagnosis for precision tumor therapy.

## Supplementary Material

Supplementary figures and tables.
