# Supplementary material for: Normalization of Tumor Vasculature by Oxygen Microbubbles with Ultrasound
Source: Theranostics. 2019 Sep 25;9(24):7370–83. doi: 10.7150/thno.37750 (PMC6831304; doi:10.7150/thno.37750)
Supplement: Supplementary file 1 — Supplementary figures and tables. [file thnov09p7370s1.pdf]

Supplementary Information

Titles: Normalization of Tumor Vasculature by Oxygen Microbubbles with Ultrasound

Authors: Yi-Ju Ho[1], Shu-Wei Chu[1], En-Chi Liao[2], Ching-Hsiang Fan[1], Hong-Lin Chan[2], Kuo-Chen Wei[3], and Chih-Kuang Yeh[1]*

[1]Department of Biomedical Engineering and Environmental Sciences, National Tsing Hua University, Hsinchu, Taiwan.

[2]Institute of Bioinformatics and Structural Biology & Department of Medical Sciences, National Tsing Hua University, Hsinchu, Taiwan.

[3]Department of Neurosurgery, Chang Gung Memorial Hospital, Taoyuan, Taiwan.

*Corresponding authors at:

Department of Biomedical Engineering and Environmental Sciences, National Tsing Hua University, No. 101, Section 2, Kuang-Fu Road, Hsinchu 30013, Taiwan.

Tel: +886-3-571-5131 ext. 34240; Fax: +886-3-571-8649

E-mail address: ckyeh@mx.nthu.edu.tw

This file includes:

Figure S1 to S5

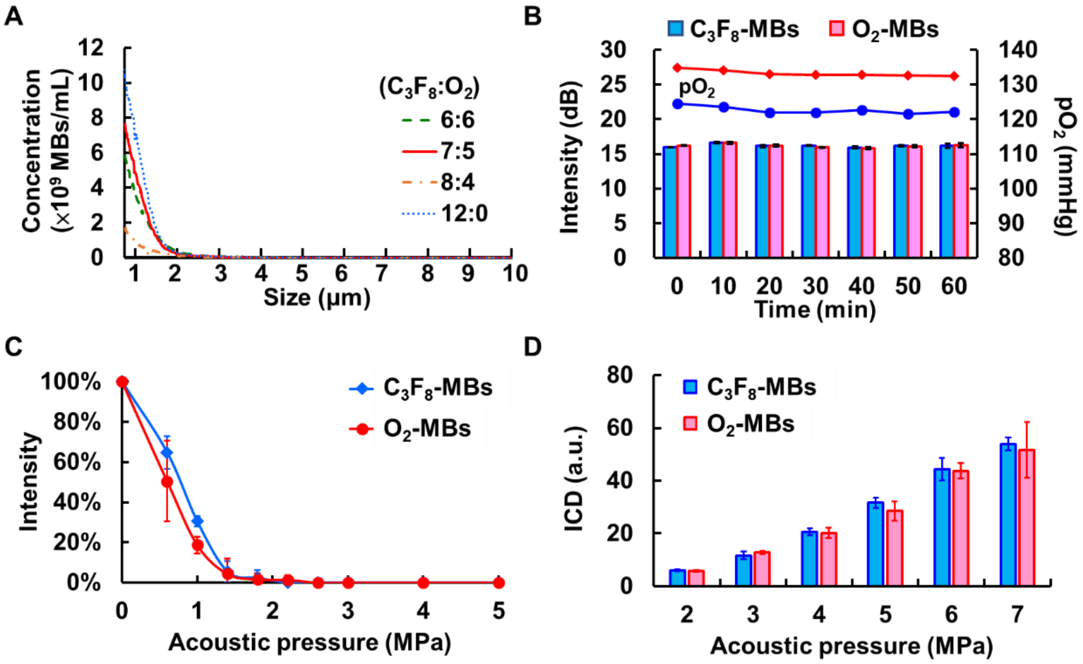

Figure S1. Physical and acoustic characteristics of C_3F_8-MBs and O_2-MBs. (A) The size distribution of O_2-MBs with various volume ratios of C_3F_8 and O_2. The optimal C_3F_8:O_2 volume ratio for O_2-MBs fabrication was 7:5. (B) The contrast enhancement of US images and pO_2 levels were measured to evaluate the stability of MBs *in vitro*. The contrast enhancement and pO_2 levels revealed no significant difference after 60 min at 37 °C in the C_3F_8-MBs and O_2-MBs groups. (C) The MBs destruction threshold under 2-MHz HIFU sonication was analyzed to determine the optimal acoustic pressure for local oxygen release. The MBs destruction at acoustic pressure of 2 MPa was 100%. (D) The ICD was determined to evaluate the possible bio-effects during MBs destruction. The ICD is directly proportional to the acoustic pressure. The physical and acoustic characteristics between C_3F_8-MBs and O_2-MBs were not significantly different. Quantitative data are presented as mean ± standard deviation and were analyzed by one-way ANOVA.

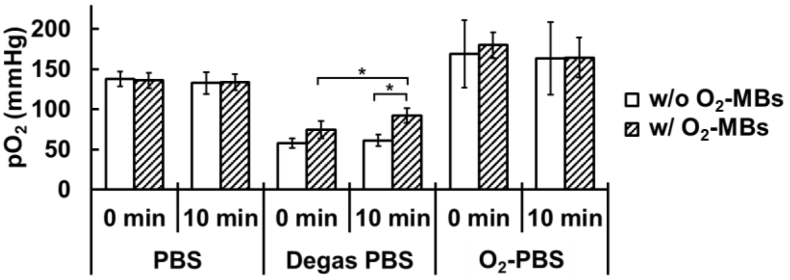

Figure S2. *In vitro* pO_2 levels of 1×10^7 O_2-MBs in the PBS, degas PBS, and O_2-PBS. The PBS was degassed for 3 min and infused O_2 for 1 min to prepare O_2-PBS. During O_2 infusion, the needle was immersed into PBS to observe the bubble production. The initial pO_2 was 138±9, 58±6, and 169±42 mmHg in the PBS, degas PBS, and O_2-PBS, respectively. In the degas PBS group, the pO_2 was significantly increased from 75±11 to 92±9 at 0 to 10 min due to the oxygen release from O_2-MBs. The results showed no significant difference over time in the PBS and O_2-PBS groups. The legends of w/o and w/ mean without O_2-MBs and with O_2-MBs, respectively.

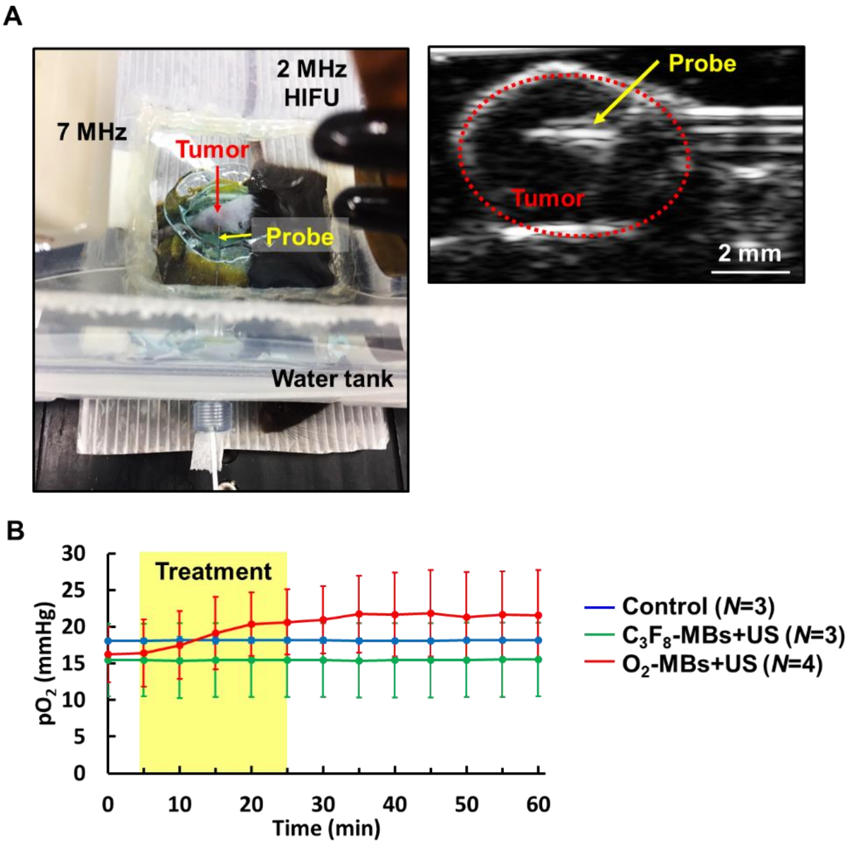

Figure S3. (A) The experimental setup of intratumoral pO_2 detection during O_2-MBs treatment. The US imaging revealed the inserted location of a fiberoptic pO_2 probe at tumor center. (B) The intratumoral pO_2 levels were 18±2 to 18±2, 15±5 to 16±5, and 16±4 to 22±6 mmHg at 0 to 60 min in the control, C_3F_8-MBs+US, and O_2-MBs+US groups, respectively. Although the results showed increment of intratumoral pO_2 levels after O_2-MBs treatment, there was no significant difference between each group due to the different initial pO_2 levels.

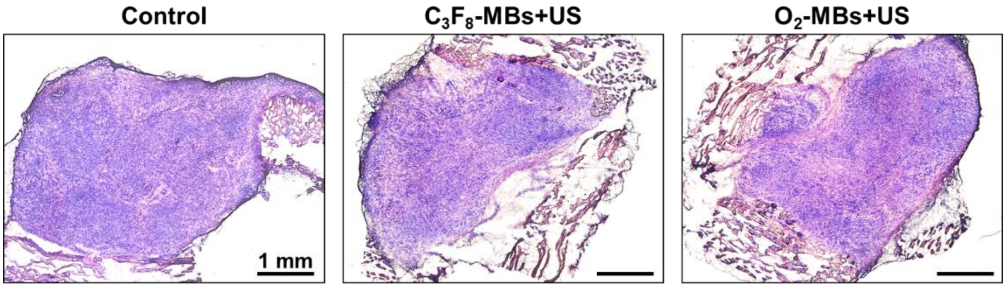

Figure S4. Histological images stained by H&E revealed intact tumor structure without hemorrhage and necrosis after O_2-MBs treatment.

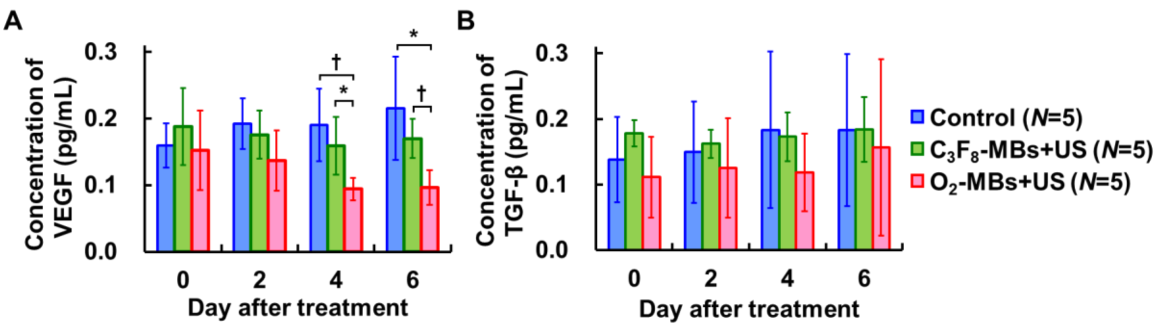

Figure S5. The variability in protein expression after O_2-MBs treatment. The concentrations of (A) VEGF and (B) TGF-β were traced over time by *in vivo* microdialysis and measured by ELISA assay. Bars are shown as means with error bars depicting the standard deviation. Data were analyzed by one-way ANOVA (* p<0.05; † p<0.01).
